# Xenograft model of heterotopic transplantation of human ovarian cortical tissue and its clinical relevance

**DOI:** 10.1530/REP-22-0114

**Published:** 2022-10-04

**Authors:** Limor Man, Nicole Lustgarten Guahmich, Eleni Kallinos, Laura Park, Richard Bodine, Nikica Zaninovic, Glenn Schattman, Zev Rosenwaks, Daylon James

**Affiliations:** 1 Ronald O. Perelman and Claudia Cohen Center for Reproductive Medicine and Infertility, Weill Cornell Medicine, New York, New York, USA; 2 Tri-Institutional Stem Cell Derivation Laboratory, Weill Cornell Medicine, New York, New York, USA; 3 Department of Obstetrics and Gynecology, Weill Cornell Medicine, New York, New York, USA

## Abstract

**In brief:**

Xenografts of human ovarian cortical tissue provide a tractable model of heterotopic autotransplantation that is used for fertility preservation in patients undergoing ablative chemo/radiotherapy. This study describes the behavior of hundreds of xenografts to establish a framework for the clinical function of ovarian cortex following autotransplantation over short- and long-term intervals.

**Abstract:**

More than 200 live births have been achieved using autotransplantation of cryopreserved ovarian cortical fragments, yet challenges remain to be addressed. Ischemia of grafted tissue undermines viability and longevity, typically requiring transplantation of multiple cortical pieces; and the dynamics of recruitment within a graft and the influence of parameters like size and patient age at the time of cryopreservation are not well-defined. Here, we describe results from a series of experiments in which we xenografted frozen/thawed human ovarian tissue (*n*  = 440) from 28 girls and women (age range 32 weeks gestational age to 46 years, median 24.3 ± 4.6). Xenografts were recovered across a broad range of intervals (1–52 weeks post-transplantation) and examined histologically to quantify follicle density and distribution. The number of antral follicles in xenografted cortical fragments correlated positively with the total follicle number and was significantly reduced with increased patient age. Within xenografts, follicles were distributed in focal clusters, similar to the native ovary, but the presence of a leading antral follicle coincided with increased proliferation of surrounding follicles. These results underscore the importance of transplanting ovarian tissue with a high density of follicles and elucidate a potential paracrine influence of leading antral follicles on neighboring follicles of earlier stages. This temporal framework for interpreting the kinetics of follicle growth/mobilization may be useful in setting expectations and guiding the parameters of clinical autotransplantation.

## Introduction

Live birth following transplantation of cryopreserved ovarian tissue was first reported in 2004 (Donnez *et al.* 2004) but has been limited in intervening years by concerns over reintroduction of malignant tissue (Bastings *et al.* 2013) and poor viability of autografted tissue following transplant (Newton *et al.* 1996, Aubard *et al.* 1999, Van Eyck *et al.* 2009, Man *et al.* 2017). Nevertheless, ovarian tissue cryopreservation (OTC) and ovarian tissue autotransplantation (OTAT) have emerged as groundbreaking options for fertility preservation in patients without an alternative. Combined with improved survival following newer treatment regimens, OTC and OTAT have changed the prospective quality of life for young girls and women, particularly now that these approaches are no longer considered experimental (asrm@asrm.org 2019).

OTAT is increasingly prevalent worldwide (Jensen *et al.* 2015, Van der Ven *et al.* 2016, Lotz *et al.* 2019, Shapira *et al.* 2020, Dolmans *et al.* 2021) – more than 200 live births have been reported in the literature. Although the number of attempted transplants remains unknown, a recent review (Dolmans *et al.* 2021) presents a multicenter series combining results from five European centers. Two hundred eighty-five patients were enrolled, with a mean age at OTC of 29.3 ± 6.2 and 34.6 ± 5.5 years at first OTAT. Eighty-nine percent of patients who underwent OTAT had a malignant disease (mainly hematological cancer and breast cancer); remaining patients had a nonmalignant disease. In this series, the majority of patients (97.5%) underwent orthotopic OTAT, with three patients receiving both orthotopic and heterotopic transplants and five patients receiving heterotopic OTAT only. Live birth rate did not differ between orthotopic sites and was between 30 and 34%. No pregnancies were reported for heterotopic sites, but the majority of OTAT in the literature has been orthotopic, with few reported attempts at heterotopic transplant (Oktay *et al.* 2001, 2004, Kim *et al.* 2009, Stern *et al.* 2014). Accordingly, a rationale for choosing orthotopic vs heterotopic sites for transplant remains unclear. Multiple studies have reviewed the outcomes and efficacy of OTAT (Meirow *et al.* 2007, Anderson *et al.* 2008, Kim 2012, Dittrich *et al.* 2015, Donnez & Dolmans 2015, Jensen *et al.* 2015); however, these have focused on clinical values and endpoints (e.g. serum hormone levels, oocyte/embryo yield, clinical pregnancy, and live birth) and provide little insight into latent follicle reserve or kinetics of follicle growth within each patient/graft.

Xenografts of ovarian tissue approximate the physiological influences present during *in vivo* folliculogenesis in a manner that is not feasible in *ex vivo* cortical strip culture. In 1998, Oktay *et al.* introduced the xenograft model to study preantral follicle growth in human ovarian cortex (Oktay *et al.* 1998), demonstrating that follicles could grow to antral stages, and many studies have employed xenografts to examine ovarian function and follicle development* in vivo* (Oktay *et al.* 1998, Weissman *et al.* 1999, Nisolle *et al.* 2000, Dath *et al.* 2010, Soleimani *et al.* 2010, Man *et al.* 2020). Multiple influences undermine the viability and productivity of ovarian tissue following OTAT, but the most detrimental is the ischemic and inflammatory microenvironment that prevails until the restoration of the vascular equilibrium in the graft (Aubard *et al.* 1999). We have previously shown that co-transplantation of human ovarian cortex with endothelial cells (ECs) confers a survival benefit to xenograft-resident follicles (Man *et al.* 2017, 2018). Although this approach is not currently suitable for clinical application, it provides a larger pool of surviving follicles capable of undergoing growth and maturation to ovulatory stages.

Although xenografts in a non-human host are subject to influences that are not present during physiological human folliculogenesis, they consistently recapitulate all stages of growth/maturation and provide an accessible platform for experimental study (Man *et al.* 2020). Here, we have performed an aggregate analysis of human cortical xenografts to define the growth trajectory of follicles over time courses, ranging from 1 to 52 weeks. Our data reveal that increased patient age correlates with reduced antral follicle production and that larger sized grafts do not promote increased antral follicle yield. Interestingly, we observed focal recruitment waves within grafts that suggested a paracrine influence of leading antral follicles on less advanced follicles in the periphery. This analysis of follicle growth kinetics following xenotransplantation to a heterotopic site sheds light on the factors that influence graft longevity and patient prognosis in a controlled replicable platform, thereby providing a resource that can help guide clinical decisions following cryopreservation and autotransplantation of cortical tissue.

## Materials and methods

### Procurement of ovarian tissue

All experiments using human tissue samples were reviewed and approved by the Ethics Committee of the Institutional Review Board (IRB) of Weill Cornell Medical College (IRB no. 0901010165). Written consent has been obtained from each patient or subject after full explanation of the purpose and nature of all procedures used. All procedures were approved by the of Weill Cornell Medical College (IRB no. 0901010165).

Tissue was obtained from three different sources:

1. Organ donors: Ovarian cortex was isolated from whole bilateral ovaries obtained from nine brain-dead organ donors, aged 32 weeks of gestation and 33 years, as a part of a collaboration with the International Institute for the Advancement of Medicine (Table 1; DOV 1–2, 4–5, 7–9, 11, and 13). The donors had no history of chemotherapy and no apparent history of endocrine or reproductive abnormalities. The ovaries were resected and placed in sterile Leibowitz’s L-15 medium (Gibco 11415-064) on ice for transport to our laboratory for processing following a cold ischemic interval of <4 h.

**Table:** Patient’s characteristics. The table shows details regarding the age of the patients, diagnosis, and past chemo, if applicable. It also elaborated the number of grafts used from each patient. Hormonal measurement, number of antral follicles and its size when available.

Patient number	Age	Past Tx/chemotherapy	History	Number Of grafts (long term (Fig. 2A))	Experiment notes	Hormonal measurement (Fig. 5A, B, C and D)					Number of antral follicles (diameter in mm)
						hE_2_, pg/mL	mFSH, ng/mL	mLH, ng/mL	hAMH, ng/mL	hTesto- sterone, ng/dL	
Patient 1	42 years	No		12							
Patient 2	19 years	12 cycles of ABVD	Hodgkin’s lymphoma	12							
Patient 3	32 years	S/p chemotherapy, prior BMT	Hodgkin’s lymphoma	6							
Patient 4				6							
Patient 5				6							
Patient 6				4							
Patient 7				4							
Patient 8				4							
Patient 9	19 years		Ewing’s Sarcoma	16 (4)	22 weeks	68.2	110.64	0.24	0.03	27.78	5 (largest 10.5)
					20 weeks						6 (largest 3.1, 2.2)
					Patient 3 in Fig. 3A						
Patient 10	17 years		Hodgkin’s stage IV	6							
Patient 11	5 years	Prior BMT	Beta-thalassemia	6							
Patient 12	6 years		Thalassemia major	2 (1)	16 weeks	84.6	65.6	0.24	0.42	19.92	6 (largest 2)
					Fig. 2E, F and G Patient 2 in Fig. 3A						
Patient 13	17 years			4	2 weeks	5	126.69	3.06	0.03	2.5	
					2 weeks	5	96.7	1.3	0.03	2.5	
Patient 14	26 years	S/p ABVD, prior BMT	Hodgkin’s lymphoma	8							
Patient 15	18 years	Doxorubicin, ara-C, vincristine, Pegapargase, MTX, and 6MP	Lymphoblastic lymphoma with recurrence	4							
Patient 16	5 years		Beta-thalassemia prior allogenic transplant	8							
Patient 17	18 years		Non-Hodgkin’s lymphoma	8							
Patient 19	17 months	TVTC	AML	6	Fig. 1B, harvest 4 weeks post-transplant						
Patient 20	46 years		Prophylactic BSO due to BRCA carrier	6							
DOV 1	26 years		Organ donor	4							
DOV 2	33 years		Organ donor	16							
DOV 4	25 years		Organ donor	95 (6;8 weeks + 17;14 weeks or more (Fig. 3D))	21 weeks	47	98.34	1.03	1.42	2.5	1 (1) 1 (2)
											5 (0.3, 0.4, 0.5, 0.5, 0.5)
											1 (0.2)
											2 (1.2, 1.3)
											2
											1
											2
											3
											2
											1
											1
					Fig. 2D Patient 4 in Fig. 3A, B, C, D						
DOV 5	29 years		Organ donor	74 (5)	4 weeks	5	96.79	3.63	0.03	2.5	
					4 weeks	5	103.24	2.94	0.03	2.5	
					14 weeks	5	104.69	4.53	0.03	2.5	
					14 weeks	61.9	45.23	0.24	0.06	2.5	1
											2 (2, 3)
											1
DOV 7	12 years		Organ donor	74 (5)	Patients 1, 5 in Fig. 3A						1
DOV 8	Neonate, 37 weeks of gestation	Anencephaly	Organ donor	3							
DOV 9	Neonate, 32 weeks of gestation	Encephalocoel	Organ donor	3							
DOV 11	28 years		Organ donor	40	EdU/CldU (Fig. 4) 11 weeks	902	13.91	0.24	2.02	27.6	1 (7)
					EdU/CldU (Fig. 4) 11 weeks	6	90.59	0.72	1.26	2.5	
DOV 13	31 years		Organ donor	3							

Tx, treatment; ABVD, adriamycin, bleomycin, vinblastine, and dacarbazine regimen; S/p, status post; BMT, bone marrow transplant; MTX, methotrexate sodium; 6MP, 6-mercaptopurine; TVTC, clofarabine with topotecan, vinorelbine, and thiotepa; AML, acute myeloid leukemia.
2. Our center’s embryology laboratory: After the IRB Committee of Weill Cornell Medical College’s approval, ovarian tissue was collected for clinical use. Upon patient’s consent, ovarian tissue was donated. In this study, we used frozen/thawed ovarian tissue that was donated by 18 deidentified patients (ages 17 months to 42 years) or by their legal guardians (Table 1; Patients 1–17, 19). The data we have on each patient are limited and appear in Table 1. No more than 10% of ovarian tissue per patient was used for research. The vials were transferred to our research laboratory on dry ice.
3. Institutional pathology department: Following informed consent, one patient underwent prophylactic bilateral salpingoophorectomy (BSO) due to being a carrier of the gene encoding breast cancer type one susceptibility protein (*BRCA1*) at the age of 46 years (Table 1; Patient 20). Fresh biopsies were transported to our laboratory in sterile saline on ice. We cryopreserved the ovarian cortex in our laboratory.

### Ovarian tissue slow freezing and rapid thawing

We bisected the ovary and removed the medulla with fine curved scissors and scalpels. Medulla tissue was removed until the remaining cortex was 1–1.5 mm thick, and this was cut into 2–3 mm wide slivers. Cortical fragments were transferred into cryogenic vials containing a freezing solution (with DMSO as cryoprotectant) and cooled in a programmable freezer, as previously described (Oktay 2001). At the end of the program, cryogenic vials were stored in liquid nitrogen (LN_2_). When ready for experimental use, vials were thawed rapidly (~100°C/min) by immersing in a 37°C water bath, followed by gradual dilution of cryoprotectant, using lower concentrations of DMSO in a stepwise manner, with gentle agitation for 5 min in each step. Thawed tissue was kept on ice in a fresh medium until transplantation.

### Ovarian cortical xenografts

All procedures were approved, and experiments were performed in accordance with the guidelines and regulations of the Institutional Animal Care and Use Committee of Weill Cornell Medicine. Twelve- to 14-week-old female NOD scid gamma (NSG) immunocompromised (Shultz *et al.* 2005) mice (Jackson Labs) were used. As previously shown (Schmidt *et al.* 2003), the density of primordial follicles varied in cortical fragments within the ovary and between ovaries, and samples lack homogeneous follicular distribution (Schmidt *et al.* 2003, Lass 2004), with follicles tending to be located in clusters and not evenly distributed throughout the tissue (Qu *et al.* 2000, Poirot *et al.* 2002, Gook *et al.* 2005). On this basis, we considered each piece as a replicate by itself. Mice were anesthetized, and a medial dorsal incision was performed. They were bilaterally ovariectomized, and ovarian cortical tissue was xenotransplanted, as previously described (Man *et al.* 2017, 2018). Briefly, cryopreserved tissue was thawed rapidly, washed of cryoprotectant, and encapsulated in fibrin that was pre-mixed with a single-cell suspension of ECs. The grafts were between 1 and 40 mm^3^, with an average of 13.1 ± 8.52 mm^3^, and a median of 12 mm^3^. Fibrin-embedded tissue was then transplanted into the mice bilaterally under the fascia of the gluteus maximus. The fascia and dorsal wall skin closed with sutures. Xenografted animals were maintained in sterile conditions until they were euthanized, and xenografts were recovered for fixation, cryosectioning, and immunohistochemical staining. Fifty-seven of 199 mice carried tissue from two patients, engrafted bilaterally. The remaining 142 mice carried two grafts from the same patient. Only 4 (of a total 199) mice were injected with human menopausal gonadotropin (HMG) to test the influence of hormonal stimulation on the development and growth of antral follicles. We used fibrin encapsulation with ECs over more traditional xenotransplantation due to the high survival rate of the follicles (Man *et al.* 2017). Importantly, the dimensions of ovarian cortical pieces varied due to the source from which they were supplied (pathology vs embryology laboratories). To optimize revascularization, we apply a ratio of 20,000 ECs per mm^3^ of cortical tissue (Man *et al.* 2017, 2018). The number of cells is calculated according to the dimensions of the graft and mixed per graft at the time of xenotransplantation.

### Endothelial cells

Human ECs were isolated from neonatal human umbilical vein endothelial cells (HUVEC), as described (Seandel *et al.* 2008). Cells were provided by Angiocrine Biosciences (San Diego, CA, USA). Cells were shipped to our laboratory and stored in LN_2_. A few days prior to xenotransplantation, cells were plated on gelatin-coated (ultrapure water with 0.1% gelatin, Millipore, catalog number ES-006-B) six-well plates, split, and passaged to achieve confluence. Cells were not passaged more than four times. ECs were cultured in advanced DMEM/F12 (catalog number 12634010) with 20% KnockOut™ Serum Replacement (catalog no. 10828028), 0.01% basic fibroblast growth factor (bFGF), 1% antibiotic-antimycotic, 1% Gibco; 200 nM l-glutamine, and 0.1% 2-mercaptoethanol.

### Encapsulation of the ovarian tissue

Human ovarian cortical tissue was encapsulated with a single-cell suspension of ECs immediately before starting the surgical procedure, as previously described (Man *et al.* 2017, 2020). Briefly, a piece of thawed human ovarian cortical tissue was placed on paraffin film within a 50 mm Petri dish. The ECs were enzymatically detached from the culture plate, counted, and placed in a microcentrifuge tube. The cells were resuspended in DMEM/F12 to a total volume of 16.8 μL, followed by resuspension with 39.2 μL cold 10 mg/mL fibrinogen solution. The solution was mixed by gentle pipetting, and 14 μL of 5 U/mL thrombin containing CaCl_2_ were added. Following gentle pipetting inside the same microcentrifuge tube, the mixture was pipetted onto ovarian tissue in a globe form, and the Petri dish was placed inside a humidified incubator at 37°C until transplantation (Man *et al.* 2018).

### Histology

Grafts were fixed with 4% paraformaldehyde (PFA) and dehydrated with sucrose 35% under optimal cutting temperature (OCT) (TissueTek) and were embedded and serially sectioned (10-μm sections). Hematoxylin and eosin (H&E) staining was used for differential follicle counts. Follicles were counted on every third section (10 μm) of the entire graft. All ovarian follicles were counted and were classified according to stage into primordial, primary, secondary, and antral follicles, following specific criteria (Gougeon 1996). Primordial follicles were characterized by one oocyte surrounded by a single layer of flattened pre-granulosa cells (GC). Intermediate follicles were counted as primary, with the oocyte surrounded by a single layer of both flattened and cuboidal GCs. Primary and secondary follicles contained an oocyte surrounded, respectively, by one single layer and at least two layers of cuboidal GCs but no antrum. Antral follicles showed the development of an antral cavity. Only normal morphologic follicles were counted. Importantly, in order not to count the same antral follicle twice, only the mid-section containing the oocyte was counted. Follicular density was calculated to obtain the number of ovarian follicles per unit volume (cubic millimeters).

### Thymidine analog incorporation

To quantify cellular proliferation within the human ovary in a way that enables resolution of cellular division within discrete temporal windows, we utilized the incorporation of consecutive thymidine analogs. Cell proliferation is commonly evaluated by antibody labeling of markers like Ki67 or phosphohistone-H3, which will indicate the proliferative state of the tissue at the time of harvest. Data regarding the dynamics of follicle development cannot be gleaned from this labeling method. Consecutive injection of two thymidine analogs, ethynyl deoxyuridine (EdU) and 5-chloro-2′-deoxyuridine (CldU), to a xenografted mouse allows resolution of cellular division within discrete temporal windows of follicle development. Two NSG mice were bilaterally xenotransplanted, with four cortical pieces, all from the same 28-year-old patient, indicated in Table 1 as DOV 11, at the dimensions of 16 mm^3^. After 11 weeks, mice were sequentially injected with EdU (100 mg/kg for 2 days, Sigma, catalog number 900584) followed by CldU (100 mg/kg for 2 days, Sigma, catalog number c6891) 24 h apart. A day later, mice were sacrificed, and the xenografts were recovered, fixed with 4% PFA overnight at 4°C, and followed by dehydration with 35% sucrose overnight at 4°C. The tissue was embedded in OCT and serially cryosectioned (10-μm sections). Cryosections were permeabilized with a blocking solution, PBS/0.1% Tween (PBST) with 5% donkey serum (Millipore) for 1 h, followed by 3× washes with PBS. For EdU staining, the Click-it Edu Cell Proliferation Kit for Imaging, Alexa Fluor 555 dye (Invitrogen) was used according to manufacturer’s instructions, followed by 3× washes in PBS and a second click reaction with 2 mM azidomethyl phenyl sulfide (Sigma), 20 mM (+)-sodium l-ascorbate (Invitrogen), and 4 mM copper sulfate (Invitrogen) in PBS for 15 min. After washing with PBS, the slides were denatured with 2M HCl (Thermo Scientific) for 30 min and quickly neutralized with two 10 min 0.1M borate washes (Thermo Scientific). Slides were washed with PBS and incubated with blocking solution for 1 h followed by incubation with rat anti-anti-bromodeoxyuridine (BrdU) antibody (Abcam) (1: 250) overnight at 4°C. The following day cryosections were incubated with secondary antibody for 1 h (1:250) Alexa Fluor 488 dye (Invitrogen), washed 3× with PBS, counterstained with DAPI, dilactate (Thermo Fisher Scientific), and mounted in Prolong Gold (Invitrogen). Images were captured using a Zeiss 710 confocal microscope.

### Ethynyl deoxyuridine and 5-chloro-2′-deoxyuridine quantification

All blocks were sectioned serially (10-μm sections) with alternating sections allocated for either H&E or immunolabeling. In enumerating Edu/CldU incorporation, every preantral follicle observed in an H&E section was separately stained in adjacent sections for EdU/CldU (total of 41 sections); for every antral follicle, five nonconsecutive sections were stained. For quantification of EdU and CldU incorporation in early follicles, boundaries were manually drawn on each follicle to obtain measurements for either the GC or stroma layer immediately surrounding the follicle. Using the Zen colocalization software, the %EdU+ or CldU+ area for each region was quantified by dividing the area of colocalization of EdU or CldU with nuclear staining (DAPI+) by the overall nuclear area (DAPI+). The area of EdU and CldU double staining was calculated by dividing the area of overlap by the total area of staining for both dyes. Only follicles with proliferating cells were considered for analysis. The percentage of EdU+, CldU+, or EdUCldU^+^ nuclei were quantified for the GC and surrounding theca/stroma (TS) of each follicle using ZEN colocalization software. In this section, the secondary follicles were broken into two subgroups: secondaries and preantral follicles. Preantral follicles are secondaries with five or more GC layers without antrum.

### Measurement of hormones

Levels of human estradiol (E_2_), testosterone, and anti-Müllerian hormone (AMH) were measured in the serum of the host mice. Blood was drawn at euthanasia, prior to harvest of the grafts, and serum was separated. These values were determined using the Roche Cobas e801 module for automated ELISA. The lower limit of detection was 5 pg/mL for E_2_, 2.5 ng/dL for testosterone, and 0.03 ng/mL for AMH.

Serum was separately sent to the Ligand Assay & Analysis Core at the University of Virginia for measurement of murine, luteinizing hormone (LH), and follicle-stimulating hormone (FSH) (EMD Millipore).

### Statistical analysis

Statistical analyses and graphs were done using Prism 9 (GraphPad). For the nonparametric comparisons, Mann–Whitney *U*-test with significance defined as *P*≤ 0.05 was used. When regression was used, a *P*-value ≤0.05 was considered significant. For assessment of cell proliferation in consecutive temporal windows of follicle development, significance was calculated using multiple unpaired *t*-tests.

**Figure 1:**
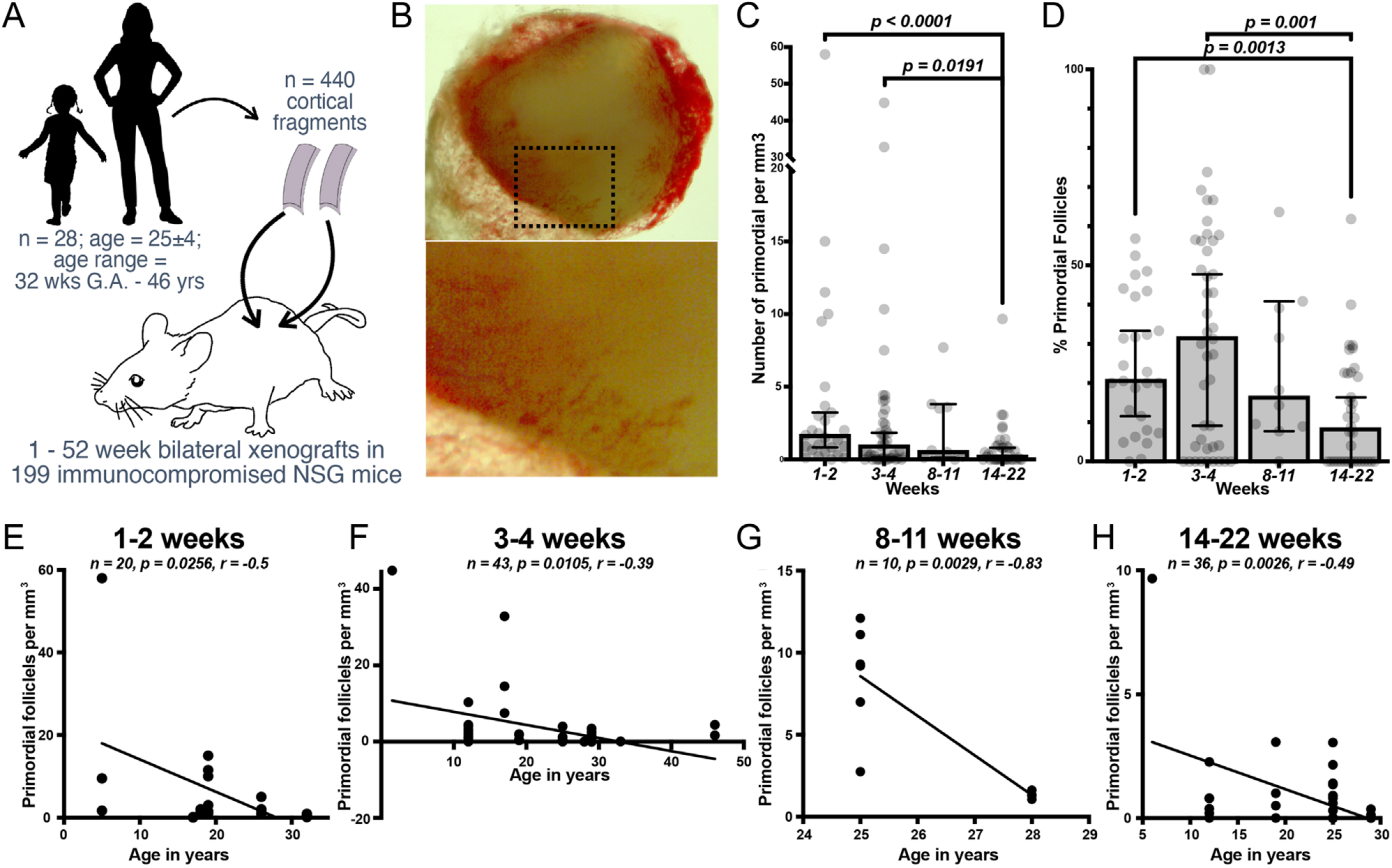
Primordial follicle retention and antral follicle output in short- and long-term ovarian cortical xenografts. (A) Schematic of the experimental design for xenotransplantation of ovarian cortical fragments from patients and organ donors. (B) A fibrin clot containing a graft harvested at 4 weeks post-transplantation (Patient 19, Table 1). (C and D) Density (C) and percentage (D) of primordial follicles retained in xenografts upon recovery between 1- and 2-, 3- and 4-, 8- and 11-, and 14- and 22-week intervals. (E, F, G, and H) The density of primordial follicles retained in xenografts at 1- to 2- (E), 3- to 4- (F), 8- to 11- (G), and 14- 22-week intervals (H) related to patient/donor age in years at the time of tissue cryopreservation. Bars in (C and D) represent median percentages of follicle sub-types, with each dot representing one replicate and error bars indicating the 95% CI.

**Figure 2:**
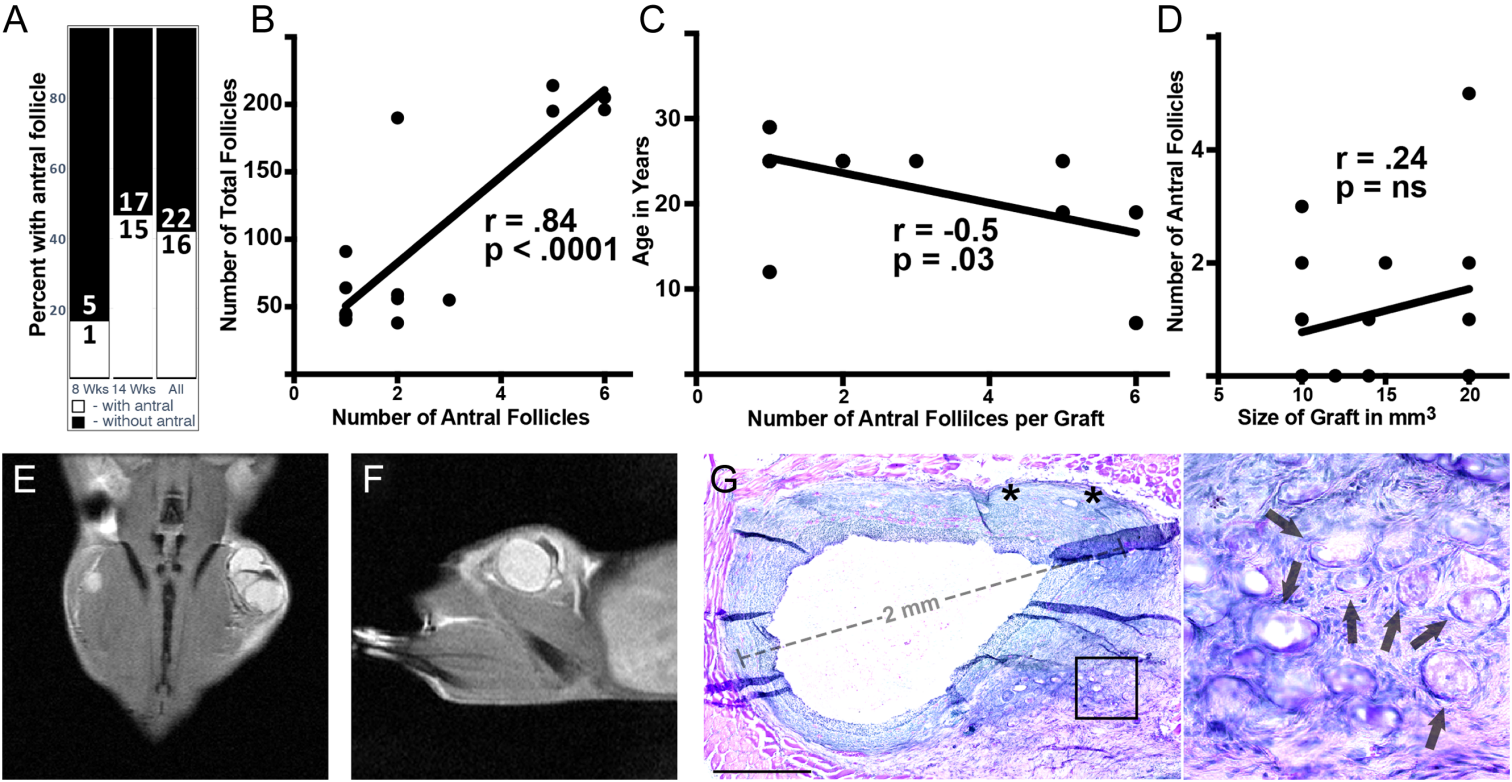
Dynamics of antral follicle growth in xenografts. (A) The number of xenografts at 8 weeks contains one or more antral follicles. (B) Relationship of the number of antral follicles to the number of total follicles in xenografts containing one or more antral follicles. (C) Number of antral follicles in xenografts relative to age in years of donor/patient at the time of tissue cryopreservation. (D) Antral follicle number (y-axis) in ovarian cortical fragments of varying sizes (x-axis); all 17 replicates are from the same patient (DOV 4, Table 1). (E and F) Mouse was xenografted with tissue from a 6-year-old donor (Patient 12, Table 1) and monitored by MRI. (E) Coronal and (F) sagittal section of the xenografts bearing mouse. (G) The mouse was sacrificed at 16 weeks post-transplantation, and grafts were harvested for histological analysis, H&E staining. Inset in G is enlarged in the associated box. Scale bar = 500 μm. Black arrows identify primordial follicles.

**Figure 3:**
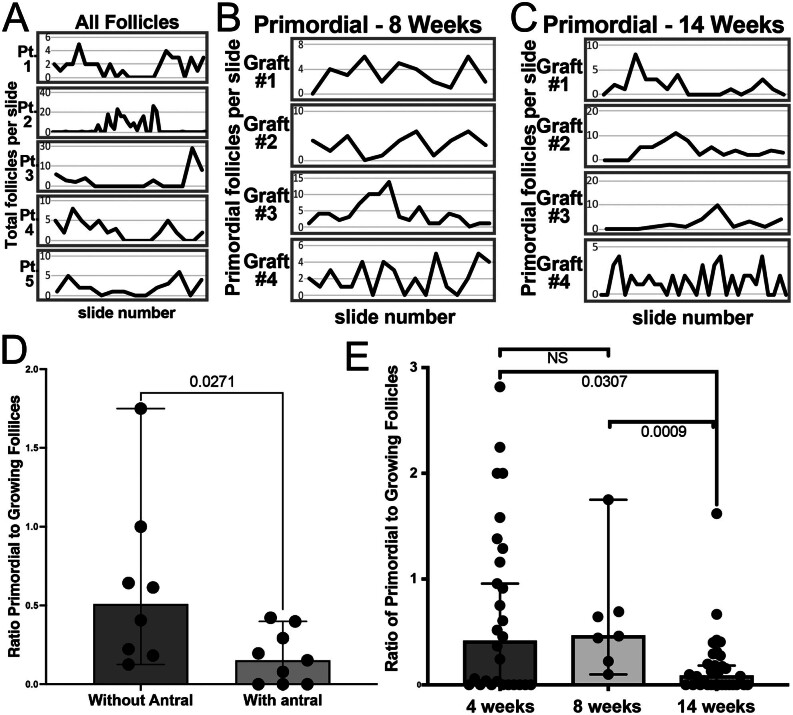
Distribution of follicles within the graft and between replicates from different patients. (A, B, and C) Graphs show the distribution of total (A) or primordial (B and C) follicles across slides encompassing the total xenograft; representative examples are shown for five grafts from four patients (Table 1) in (A) and across four recovered xenografts after 8 (B) and 14 (C) weeks from the same patient (DOV 4, Table 1). (D) The primordial to growing follicle ratio in grafts where no antral follicles were detected compared to grafts with one or more antral follicles. (E) The primordial to growing follicle ratio was measured in three different time points: 4, 8, and 14 weeks. Bars in D and E represent median ratios, with each dot representing one replicate and error bars indicating the 95% CI.

## Results

### Negative correlation between density of primordial follicles and graft duration or patient age

We used cryopreserved cortical fragments originally obtained from a subset of patients/donors in our repository (*n* =  28, median age = 25 ± 4, range = gestational age 32 weeks to 46 years; Table 1), transplanting a total of 440 fragments in 199 immunocompromised (NSG) mice (Fig. 1A). For all xenografts, we applied an EC co-transplantation strategy previously developed by our group (Man *et al.* 2017, 2018), resulting in accelerated and improved vascular perfusion (Fig. 1B and Table 1). From the long-term group, although 17 long-term xenografts were recovered for transcriptomic sequencing, thus requiring enzymatic digestion (Man *et al.* 2020), the majority of xenografts were recovered for histologic quantification of follicle volume and distribution and revealed a relative decrease in both the density (Fig. 1C) and percentage (Fig. 1D) of primordial follicles in long-term (14-22 weeks) xenografts relative to earlier (1–2 and 3-4 weeks) intervals. Sub-analysis of each interval (Fig. 1E, F, G and H) showed a reduced density of primordial follicles in tissue from women that were older at the time of OTC, although primordial follicles remained detectable in the long-term grafts from older patients. Importantly, we observed a range of densities at all ages and endpoints, thereby underscoring the importance of an individual assessment of ovarian reserve in each patient before tissue harvest for cryopreservation.

### Timing and trends in antral follicle development

A primary endpoint of OTAT is the cultivation of antral follicles that yield an oocyte capable of fertilization. To define the timing of antral follicle development, we analyzed 38 xenografts, six at 8 weeks and 32 at 14 weeks or later. One of six xenografts contained at least 1 antral follicle at 8 weeks and 15 of 32 xenografts contained at least 1 antral follicle at 14 weeks or later (Fig. 2A), indicating a moderate correlation between graft duration in weeks and antral follicle yield (*n*  = 38, r = 0.51). When present, antral follicle yield was strongly correlated with total follicle number (*n*  = 16, r = 0.84, Fig. 2B). Total follicles present exhibited a moderate inverse correlation with age of the patient/donor at the time of OTC (*n*  = 16, r = –0.42), and antral follicle production was significantly reduced (*n*  = 16, r = –0.5, *P* < 0.05) with patient/donor age (Fig. 2C). Importantly, a sub-analysis of follicle number as it relates to size of xenografts >8 weeks revealed no correlation between total (*n*  = 38, r = 0.009, *P* = ns) or antral (*n*  = 38, r = –0.13) follicles. In support of this, a sub-analysis of 17 replicates from a single 25-year-old donor (DOV 4, Table 1) also showed no significant correlation (*n*  = 17, r = 0.24, *P* = ns, Fig. 2D) between antral follicle number and graft size.

Although many patients have undergone OTC for childhood cancer and malignancies, only two cases have reported live birth following OTAT of tissue cryopreserved during or before the onset of menarche (Demeestere *et al.* 2015, Matthews *et al.* 2018). To measure the capacity of prepubertal tissue to generate antral follicles, we xenografted tissue from a thalassemia major patient who was 6 years old at the time of cryopreservation (Patient 12, Table 1) (Fig. 2E, F and G). We transplanted a volume of 6 mm^3^ frozen/thawed ovarian cortical tissue and monitored the development of follicles by serial MRI (Fig. 2E and F). Antral follicles were detected at 14 weeks post-transplantation and harvested at 16 weeks. Of a total of 196 follicles in these grafts (30% primordial), we counted six at antral stage (Fig. 2G). Assay of host serum revealed human E_2_ (84.6 pg/mL), human AMH (0.42 ng/mL), and human testosterone (19.92 ng/dL), presumably xenograft derived, as well as low levels of mouse FSH 65.6 ng/mL and undetectable mouse LH<0.24 ng/mL. While this represents tissue from a single patient, it definitively demonstrates the capacity for tissue cryopreserved as early as 6 years of age to generate antral follicles following heterotopic xenotransplantation.

### Paracrine influence of antral follicles on the pre-antral pool

In the native ovary, quiescent primordial follicles are distributed within the cortex, moving inward toward the medulla as they are activated and mature (Woodruff & Shea 2011). Follicle growth in a typical natural cycle is controlled via local (intra-ovarian) and systemic (inter-ovarian) cues that govern the emergence, and eventual ovulation, of a single lead follicle. Following the transplantation of the cryopreserved ovarian cortex, more advanced follicles do not survive freeze/thaw/transplant, resulting in a ‘reset’ of the surviving follicular pool. Enumeration of all (Fig. 3A and Table 1) or exclusively primordial (Fig. 3B, C and Table 1) follicles in cryosections of recovered xenografts showed an uneven distribution between patients, in a cluster pattern, across ages (Fig. 3A), and within the same patient at 8 (Fig. 3B), and 14 weeks post-transplantation (Fig. 3C). To identify characteristics of cortical fragments that may influence the generation of antral follicles, we divided 17 replicates originating from a single patient, DOV 4 (Fig. 3D and Table 1), into two groups – those that did not (*n* = 8) or did (*n* = 9) give rise to one or more antral follicle. Interestingly, we observed a significant difference between the median ratios of primordial to growing follicles (0.51 vs 0.15, *P* < 0.05; Fig. 3D) between these groups. There was no significant difference in the size of grafts (12.25 ± 4.83 mm^3^ vs 13.67 ± 5.1 mm^3^) or the number of total follicles per mm^3^ (average 7.38 ± 2.98 vs 5.9 ± 2.49) between groups. To determine whether the presence of antral follicles merely reflects a cluster of growing follicles or alternatively acts as a driver of follicle activation and growth, we analyzed the change of the ratio of primordial to growing follicles over three time points: 4 weeks (*n* = 28), 8 weeks (*n* = 7), and 14 weeks (*n* = 33). Between 4 and 8 weeks, there was no difference in the ratio median of 0.41 at 4 weeks, compared to 0.46 at 8 weeks. At 14 weeks, the ratio dropped significantly to 0.08 (Fig. 3E and Table 2).

**Table 2:** Differential counting of follicles. The table shows the differential counting of grafts, including patients’ details.

Number	Patient number	Primordial	Primary	Secondary	Antral	Primordial to growing ratio
4 weeks						
1	DOV 5	0	20	32		0
2	DOV 5	63	68	36		0.60576923
3	DOV 5	2	30	22		0.03846154
4	DOV 5	55	92	58		0.36666667
5	DOV 5	0	37	30		0
6	DOV 5	15	50	12		0.24193548
7	DOV 5	15	26	3		0.51724138
8	DOV 5	5	65	20		0.05882353
9	DOV 7	4	2	0		2
10	DOV 7	26	11	2		2
11	DOV 7	31	9	2		2.81818182
12	DOV 7	3	4	0		0.75
13	DOV 7	3	0	0		0
14	DOV 7	0	7	3		0
15	DOV 7	155	51	18		2.24637681
16	DOV 7	29	16	5		1.38095238
17	DOV 5	0	11	13		0
18	DOV 5	0	5	7		0
19	DOV 5	1	9	21		0.03333333
20	DOV 5	0	17	48		0
21	DOV 7	22	18	5		0.95652174
22	DOV 7	29	17	8		1.16
23	DOV 7	49	33	5		1.28947368
24	DOV 7	53	46	12		0.9137931
25	DOV 11	0	3	4		0
26	DOV 11	0	3	14		0
27	Patient 19	5	10	1		0.45454545
28	DOV 4	538	282	58		1.58235294
8 weeks						
1	Patient 7	46	57	47	0	0.44230769
2	DOV 4	4	18	0	0	0.22222222
3	DOV 4	5	31	18	2	0.09803922
4	DOV 4	36	40	16	0	0.64285714
5	DOV 4	35	57	19	0	0.46052632
6	DOV 4	77	34	10	0	1.75
7	DOV 4	38	38	17	0	0.69090909
14 weeks						
1	DOV 4	27	46	17	1	0.421875
2	Patient 9	3	10	21	0	0.09677419
3	Patient 9	6	26	13	0	0.15384615
4	DOV 4	28	54	15	0	0.4057971
5	DOV 4	43	86	59	2	0.29251701
6	DOV 4	18	112	32	0	0.125
7	DOV 4	61	102	46	5	0.39869281
8	DOV 7	0	0	0	0	0
9	DOV 7	4	18	5	0	0.17391304
10	DOV 7	68	32	10	0	1.61904762
11	DOV 7	16	8	15	1	0.66666667
12	DOV 7	15	32	16	0	0.3125
13	DOV 7	19	39	30	0	0.27536232
14	DOV 5	1	15	9	0	0.04166667
15	DOV 5	0	24	17	0	0
16	DOV 5	5	15	23	1	0.12820513
17	DOV 5	0	0	0	0	0
18	DOV 5	0	12	22	0	0
19	DOV 5	10	22	12	0	0.29411765
20	DOV 4	9	33	10	3	0.19565217
21	DOV 4	8	34	10	0	0.18181818
22	DOV 4	3	24	13	1	0.07894737
23	DOV 4	0	40	17	2	0
24	DOV 4	0	38	16	2	0
25	DOV 4	0	23	20	1	0
26	DOV 4	0	0	1	0	0
27	DOV 4	0	1	0	0	0
28	DOV 4	6	56	18	0	0.08108108
29	DOV 4	0	11	1	0	0
30	DOV 4	0	1	0	0	0
31	DOV 4	0	34	14	0	0
32	DOV 4	0	39	24	1	0
33	DOV 4	0	45	11	0	0

**Figure 4:**
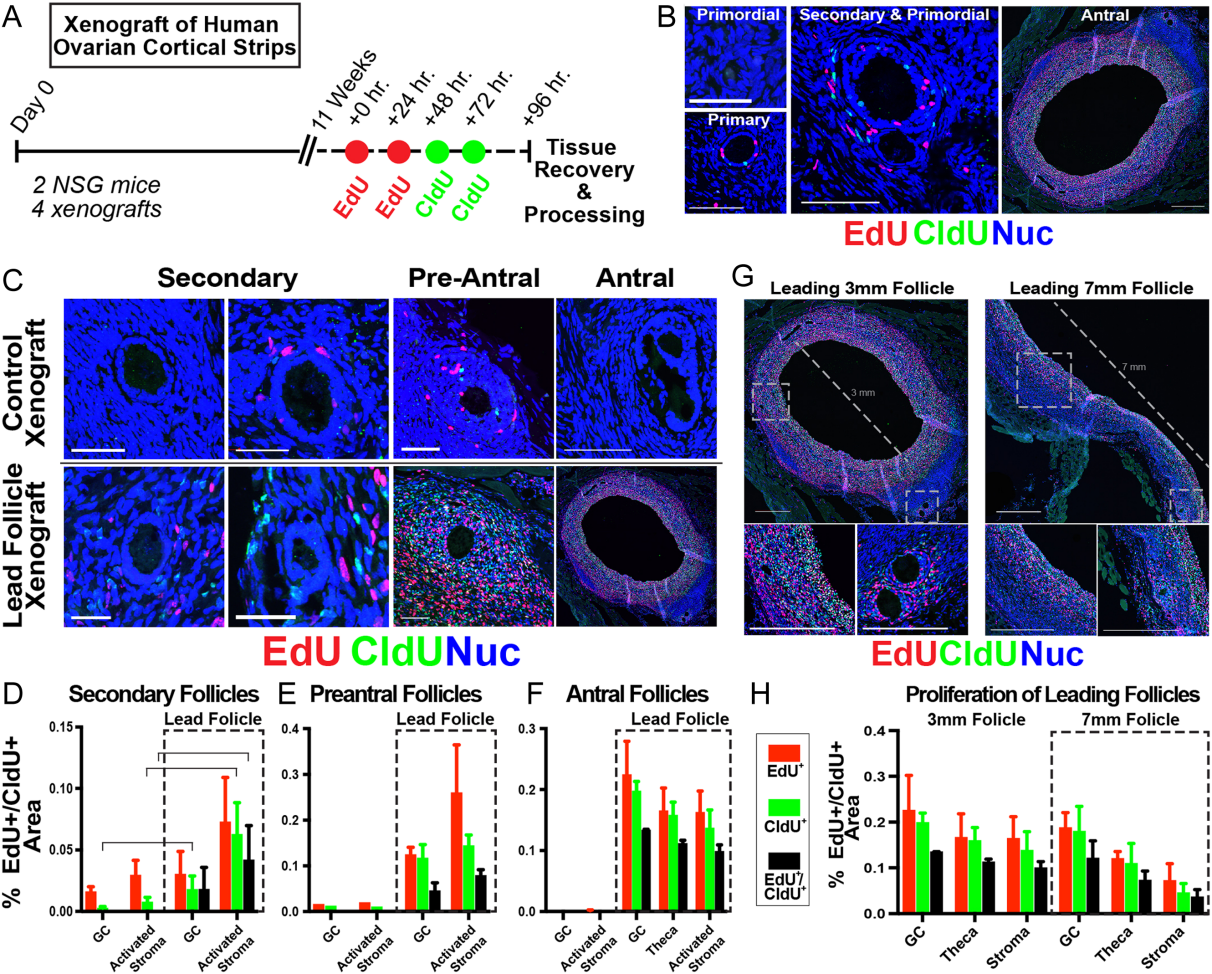
Assessment of cell proliferation in consecutive temporal windows of follicle development. (A) Schematic of the experimental approach for sequential labeling with thymidine analogs; red and green dots indicate times of EdU and CldU injections, respectively. (B) Labeling detected proliferative cells in growing follicles of all stages (primary to antral). (C, D, E, F, G, and H) Quantification of proliferative cells within the GC and activated stroma compartment of follicles. (C, D, E, and F) Follicles from xenografts containing leading follicles showed a significant increase in GC and TS proliferation when compared to follicles in grafts without leading antral follicles, observed at secondary (D) (up to four layers of GCs), preantral (E) (five layers or more of GCs), and antral stages (F), as measured by incorporation of EdU, CldU, or both. (G and H) ST compartment proliferation decrease with increased size approaching ovulatory competence. (B, C and G) Scale bars in (B and C) are 50 μm; in (G), scale bars are 500 μm and 100 μm in the insets.

To provide a quantitative measure of follicle growth in the presence/absence of antral follicles, we used consecutive injections of two thymidine analogs to identify cells that were proliferating in discrete temporal windows (Fig. 4A and B). Beginning at the secondary stage, follicles (*n* = 17) from xenografts containing a leading follicle (>3 mm, *n* = 2 xenografts) showed a significant increase in GC (*P* < 0.05) and TS (*P* < 0.05) proliferation when compared to follicles (*n* = 17) in grafts without leading antral follicles (*n* = 2) (Fig. 4C and D). Preantral (*n* = 5) (Fig. 4C and E) and small antral (*n* = 2) follicles (Fig. 4C and F) in the leading follicle condition showed an increased presence of rapidly dividing, double-positive EdU**^+^**CldU**^+^** cells (*P* < 0.05), while in the control (non-leading follicle) condition, the few identified preantral (*n* = 1) and antral (*n* = 1) follicles exhibited no or limited proliferation, respectively (Fig. 4D, E and F). Comparison of the leading follicles, a 3-mm antral and a 7-mm periovulatory follicle, revealed that while GC proliferation is similar between these stages, proliferation within the TS compartment was reduced with increased size (Fig. 4G and H).

### Host serum values as a proxy measure of antral follicle development

**Figure 5:**
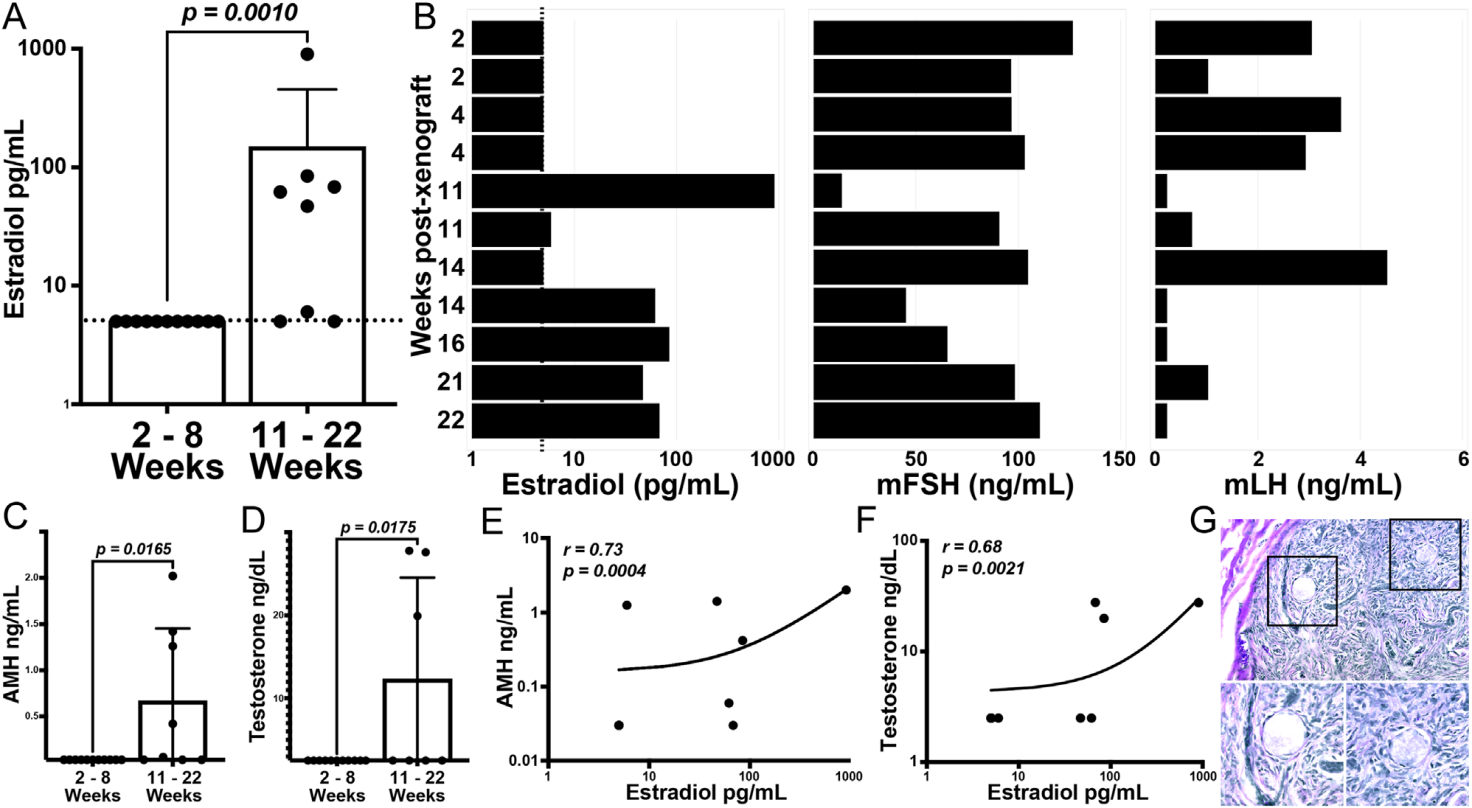
Reproductive hormones/steroids in host serum reflect follicle activity in xenografts. (A) E_2_ was measured in the serum of animals bearing xenografts for short (2–8 weeks) and long (11–22 weeks) intervals. (B) Serum human E_2_, mFSH, and mLH were measured in a subset of mice bearing xenografts for short- and long-term intervals. (C, D, E, and F) AMH (C and E) and testosterone (D and F) were measured in the serum of animals bearing xenografts for short- and long-term intervals; levels of AMH (E) and testosterone (F) relative to E_2_ are shown for samples that had detectable levels. (G) H&E micrograph from neonatal (32 weeks gestational age) ovarian tissue that was xenografted for 52 weeks; magnified stroke boxes show primordial follicles. Bars in (A) represent median E_2_ levels (pg/mL) in the serum, with each dot representing one replicate and error bars indicating the 95% CI. Bars in (C) represent median AMH levels (ng/mL) in the serum, with each dot representing one replicate and error bars indicating the 95% CI. Bars in (D) represent median testosterone levels (ng/dL) in the serum, with each dot representing one replicate and error bars indicating the 95% CI.

To determine whether serum values provide a reliable surrogate for the emergence of more advanced-stage follicles, we collected host blood at euthanasia and measured human E_2_, testosterone, and AMH and mouse FSH and LH. We compared these factors in mice bearing short- (2–8 weeks, *n*  = 11) and long-term (11–22 weeks, *n*  = 8) xenografts (Fig. 5A, B, C and D); grafts were included in the analysis regardless of whether they contained antral follicles. Estradiol was significantly increased (*P*  <0.05) in the serum of mice bearing long-term xenografts (Fig. 5A and Table 1), and this increase coincided with reduced levels of mouse FSH/LH (Fig. 5B and Table 1). Mice bearing long-term xenografts also had significantly higher serum values of AMH (Fig. 5C and Table 1) and testosterone (Fig. 5D and Table 1) that correlated with increased E_2_ (Fig. 5E, F and Table 1). All host mice were ovariectomized in advance of xenograft; hence serum values reflect graft-derived factors (human E_2_, testosterone, and AMH) and host pituitary-derived hormones (murine FSH and LH). Notably, one mouse that carried a xenograft for a year showed positive serum AMH (0.16 ng/mL) and retention of residual primordial follicles within the xenograft (Fig. 5G).

## Discussion

OTAT is a proven means of safeguarding fertility for young girls and women facing gonadotoxic therapies, yet a deeper understanding of the dynamics post grafting is imperative to optimizing the chances of positive outcome for each patient. Autografted ovarian tissue is subjected to a unique environment following transplantation that disrupts the equilibrium present in the native ovary. Grafts are transplanted with no end-to-end anastomosis; therefore ischemia has a critical deleterious influence during the first hours and days post-transplantation (Van Eyck *et al.* 2009). Rigorous analysis of transplanted cortical tissue in the clinical setting is impractical because patient tissue is scarce, and autografts are typically not examined histologically following the recovery of oocytes. Xenograft of human ovarian tissue in mice, by contrast, provides a means of validating the latent reproductive potential of ovarian tissue following cryopreservation. Here, we have extended the use of xenografts to investigate the dynamics of follicular growth in the weeks and months after heterotopic transplantation. To our knowledge, this is the largest series of xenotransplanted human ovarian cortical tissue published in the literature, and we show the highest number of antral follicles produced from human xenografts arising from the largest age range of patients (Dath *et al.* 2010, Ruan *et al.* 2019). We elaborate on the use of ovarian tissue arising from 28 patients and a total of 440 replicates and perform a large-scale analysis to delineate the progressive activation of the primordial follicle reserve and the emergence of antral stage follicles over long-term intervals. The latest time point we examined was 1 year, with the finding of residual primordial follicle in the graft, which suggests that this model can additionally be utilized for long-term follow-up of secondary recruitment waves within xenografts.

A major benefit of the xenograft model we have developed is expedited revascularization afforded by co-transplanted ECs and the resultant preservation of a higher proportion of follicles (Man *et al.* 2017). In our model, we encapsulate human ovarian tissue with a single-cell suspension of ECs in a fibrin clot. The proximity of the ECs to the graft accelerates the revascularization and salvages a robust volume of the transplanted tissue. While co-transplantation of ECs is not employed for clinical OTAT, our transplantation model faithfully recapitulates the revascularization process following transplantation to a heterotopic site. We have previously performed an analysis using both bulk and single-cell RNA sequencing to distinguish the transcriptional signature of GC and TS cells that were prospectively isolated from human antral follicles derived from xenografts or native ovaries. Although multiple factors are differentially expressed in follicles of xenograft vs ovarian origin (Man *et al.* 2020), the growth and maturation of follicles in xenografts mirrors that of the native ovary (Man *et al.* 2022) and they are capable of advancing to ovulatory stages (Man *et al.* 2017). We have separately utilized exogenous ECs to demonstrate that paracrine delivery of AMH to xenograft mitigates primordial follicle activation in the short term (Man *et al.* 2017) and drives premature luteinization of antral stage follicles over long-term intervals (Man *et al.* 2022). Also, we have employed this model to demonstrate that exposure to ectopic insulin-like growth factor 1 (IGF-1) from exogenous ECs increases the growth of early-stage follicles (Man *et al.* 2021). While these studies illustrate the potential for co-transplanted ECs in the xenograft system to interrogate the influence of secreted factors on follicle development, the 440 xenografts described in this study were co-transplanted with ECs not engineered to secrete specific factors. This approach faces many hurdles to clinical application, yet the experimental use of ECs in xenografts expands the pool of retained follicles, thereby affording a more robust model of human folliculogenesis.

Despite the benefit afforded by co-transplantation of ECs, xenografts show a progressive reduction of the primordial follicle pool with increasing graft duration. This aligns with previous studies that have demonstrated accelerated activation of the follicle pool following freeze/thaw and transplant (Dolmans *et al.* 2007). It is well established that ovarian reserve declines with age (Hansen *et al.* 2008, Broekmans *et al.* 2009), and this is supported by our data (Fig. 1E, F, G and H) – patient/donor age at the time of OTC correlated negatively with antral follicle output (Fig. 2C). Indeed, the chance for a positive outcome following OTC and OTAT derives from balancing the initial volume of primordial follicles with the inevitable loss from processing, freezing, thawing, and transplanting tissue, including ischemia–reperfusion injury and inadequate revascularization (Newton *et al.* 1996, Roness & Meirow 2019). Accordingly, an age cutoff for cryopreservation was suggested with a maximal threshold between 35 (Wallace *et al.* 2014) and 40 (Oktay 2002, Kim *et al.* 2012, Meirow *et al.* 2016) years. Our data support this cutoff; however, antral follicle production was noted from tissue of a broad age range. This suggests that OTAT should not be limited to young patients but instead be personalized to account for individual patient’s ovarian reserve; an assessment should be performed to evaluate ovarian reserve, medical background, underlying disease, medications, past procedures, any history of gonadotoxic exposure, environmental exposure, and perhaps genetic background.

While a maximal age threshold for OTC and OTAT has been suggested, it is unclear whether there is a minimum threshold for cryopreserving ovaries from pediatric patients due to a lack of documented clinical attempts. Poirot *et al.* published the largest series of young patients, including girls and adolescents younger than 15 years. In the series, about 20% of the patients passed away and the utilization rate was low – only three patients requested autotransplantation (Poirot *et al.* 2019). To date, the earliest OTC/OTAT that resulted in live birth was in a patient who was 9 years old at the time of tissue retrieval, although ovarian cortical fragments in this patient were transplanted to the remaining ovary (Matthews *et al.* 2018). Using heterotopic xenotransplantation, we did not find a difference in the morphology of follicles between grafts from patients of different ages. Indeed, following long-term xenotransplant of ovarian tissue from a 6-year-old patient (Patient 12, Table 1), we achieved multiple antral follicles (Fig. 2E, F and G), thereby supporting the possibility of antrum development following heterotopic xenotransplantation of tissue from a prepubertal girl.

The size and thickness of cortical fragments in advance of OTC also contributes to the behavior of grafts. Thinning the cortical sliver during processing is a common practice based on the idea that it improves the penetrance of cryoprotectant before freezing while also accommodating the passive diffusion limit of oxygen in nonvascularized tissue (100–200 um) (Krogh 1919). Yet surprisingly, an ovary xenograft study that directly compared the impact of graft dimensions found that reducing graft thickness does not significantly increase revascularization of the graft (Gavish *et al.* 2015). Moreover, increased manipulation to reduce graft thickness had the additional adverse effect of increasing follicle activation, resulting in greater loss of dormant follicles. Similar activation of follicles in grafted tissue of small dimensions was also highlighted by Kawamura *et al.* (2013). Ultimately, the size of cortical fragments is governed by practical considerations: for example, if using a laparoscope for OTAT, cortical fragments must be large enough to handle and manipulate; transplantation technique and sites are variable, with most groups transplanting into orthotopic sites in the remaining ovary (Meirow *et al.* 2005, Donnez *et al.* 2006, Jadoul *et al.* 2017), ovarian fossa (Donnez *et al.* 2004), or within a peritoneal pocket (Donnez *et al.* 2006). Some approaches employ synthetic material to cover the grafts (Dolmans *et al.* 2021), while others assemble fragments into a ‘quilt’ (Stoop *et al.* 2014). Heterotopic sites have been used either by manual threading of cortical pieces on a suture placed in a peritoneal tunnel (Gook *et al.* 2021) or as individual pieces (Sonmezer & Oktay 2010). Collectively, these considerations must all be taken into account when deciding the dimensions and processing/transplantation methods.

The size of cortical fragments may also impact viability and longevity and varies widely among centers (sizes from 2 mm^3^ to 60 mm^3^ (Shapira *et al.* 2020)). To address the relevance of graft size, we performed a sub-analysis using multiple pieces of ovarian tissue from the same patient and found no correlation between the dimensions and the number of antral follicles yielded (Fig. 2D). Further support for the cryopreservation of smaller fragments relates to greater flexibility regarding the volume of thawed tissue that will be transplanted back per procedure. In a large series of transplantations of frozen/thawed ovarian tissue, the longevity of transplanted tissue was shown to be variable, but many patients have experienced several years of sustained endocrine function following autotransplantation (Jensen *et al.* 2015). This provides a rationale for using of a larger proportion of cortical tissue – on average, 45% of one ovary was transplanted during initial OTAT procedures (Andersen *et al.* 2012). Yet the failure of larger fragments in our analysis to generate significantly more antral follicles suggests that transplantation of multiple smaller fragments may be favorable for increased oocyte yield. Optimally, cortical fragments can be sliced thinly enough to visualize follicle density using a stereomicroscope (Soleimani *et al.* 2006), thereby enabling prospective assessment of tissue undergoing cryopreservation.

Assessment of cell division during consecutive temporal windows of follicle development suggested an interfollicular paracrine signaling relationship that has not been described. It has previously been shown that growth of follicles is observed in ‘hotspots’ within the human ovary (Schmidt *et al.* 2003, Lass 2004). We observed that follicles were randomly distributed in cortical fragments (Fig. 3A), proliferation of stromal cells preceded that of GCs in activated follicles (Fig. 4C), and presence of a leading antral follicle coincided with proliferation of surrounding pre-antral follicles (Fig. 4D, E, F and G). The observation that lead follicles influenced the activity of surrounding small follicles was unexpected, as expression of AMH from growing follicles has been shown to suppress growth and activation at primordial and early-stage follicles (Durlinger *et al.* 2002). However, peak AMH expression occurs in growing follicles ≤4 mm (Weenen *et al.* 2004), and we have previously demonstrated an accelerated pace of follicle maturation in xenografts (Man *et al.* 2020); hence the suppressive input of AMH may have been minimal in xenografts containing leading antral follicles (3 mm and 7 mm). Additionally, the behavior of tissue-resident follicles in xenografts may not accurately reflect follicle growth dynamics in the native ovary. Nevertheless, the observation of increased growth in the periphery of lead follicles may point toward novel paracrine influences from follicles and/or ovarian stroma and could be clinically relevant in formulating strategies for OTAT in patients.

Despite the ischemic/inflammatory environment in ovarian tissue grafts post-transplant, primordial and small preantral follicles have a survival advantage due to reduced metabolic demand. While the high volume of transplanted tissue and limitations of our methodological approach precluded counting of ovarian follicles before engraftment, the high replicate number increased the power of the study. At early time points (4 and 8 weeks following the transplantation), we observed a consistent ratio of primordial to growing follicles (Fig. 3E) that was reduced at the 14-week time point, aligning with the emergence of antral follicles. Notably, when comparing two groups of grafts from the same patient with compatible size and total follicle density, between 8 and 14 weeks post-transplantation, xenografts that generated observable antral follicles had a significantly lower ratio of primordial to growing follicles compared to those without antral follicles (Fig. 3D). While we have observed this phenomenon only within a limited range of xenografts that may not accurately reflect the physiological steady state in the human ovary, it may still be relevant to heterotopic OTAT, as lead follicles may similarly drive increased activation and thereby limit the longevity of grafts. Indeed, we showed that the ratio of primordial to growing follicles decreased in the presence of antral follicles. Whether this increase of activity in the proximity of lead follicles will result in a greater or lesser number of mature follicles in subsequent waves remains an open question of considerable clinical relevance.

The xenograft model we have employed provides a robust means of determining follicle growth kinetics. We have shown successful engraftment of prepubertal tissue from neonates, toddlers, and prepubertal girls and were able to validate the longevity of tissue out to 1-year post-xenograft in an NSG mouse. Utilizing our EC co-transplantation model enables higher survival and higher yield of antral follicles, thereby providing a reliable source of follicles available for research and enabling utilization of smaller fragments of ovarian cortex to generate productive grafts. Notably, antral follicles can grow to large dimensions; the largest follicle we harvested was at a diameter of 10.5 mm (Table 1). Given the dramatic change in tissue volume coinciding with antrum formation, an important consideration for ovarian cortical xenografts is the selection of the transplantation site. The gluteus maximus muscle provides an appropriate space for development with less growth restriction compared to other sites, like the kidney capsule or the ovarian bursa, thereby enabling transplantation of relatively large dimensions of engrafted ovarian tissue. The gluteus maximus is discreet and enclosed by the fascia, preventing graft translocation that may occur in abdominal transplantation sites; and due to its superficial location, the xenografts can be visualized/palpated through the mouse’s fur with no need for anesthesia or imaging. Alternatively, emergence of antral follicles and their progressive growth can be visualized using MRI when targeting a specific antral follicle size for harvest.

As an alternative to direct visualization of follicle growth, the progression of follicle development is also reflected by serum hormone levels. In this study, we present the results of transplantation to 199 mice, with only four of those mice injected with hHMG to test the yield of stimulation on the development and growth of antral follicles. No advantage was noted with this treatment, so it was discontinued. Interestingly, monitoring of xenograft host hormonal level revealed a drop in the levels of mFSH and mLH in parallel with the emergence of antral follicles (Fig. 5 and Table 1). These results suggest crosstalk between xenograft-resident follicles and the host hypothalamic/pituitary axis. Indeed, xenograft/host crosstalk may also account for the failure of exogenous hHMG to augment follicle growth – ovariectomy of all hosts results in a high baseline level of endogenous gonadotropins that may stimulate follicle growth within the xenograft.

The observation that multiple antral follicles emerge in a single xenograft may mimic the process of cohort requirement before the selection of a dominant follicle. The ischemic/inflammatory influence in the acute phase following xenograft preferentially ablates more advanced follicles, thereby ‘resetting’ the cohort of follicles to a globally early stage. The dynamics presented in our study affirm that selection for dominance occurs at later stages of antral development, and this model provides a means of interrogating the mechanisms of follicle selection. However, the ability of the cortical xenograft model to serve as a proxy for graft behavior in the context of heterotopic OTAT is limited. The volume of xenografted fragments in this study was dramatically smaller than what is used in common practice for OTAT (Gellert *et al.* 2018) and thus might affect the longevity of the grafts. All xenografts described in this study were co-transplanted with ECs; thus, values may represent optimized survival rates that do not accurately reflect outcomes following clinical OTAT. Finally, our previous analysis (Man *et al.* 2020) revealed subtle differences in the molecular signature of cells derived from native vs xenografted antral follicles. However, the relevance of these molecular differences to antral follicle development and the quality/competence of oocytes remains to be determined, as the extra-species context of xenografts is not akin to heterotopic OTAT in patients. Our results shed light on follicle retention, activation, and growth dynamics following heterotopic transplant and emphasize the importance of carefully assessing tissue before OTC and monitoring patients closely after OTAT. As more patients who have undergone OTC in their youth begin to consider family building, a rigorous and expansive accounting in both experimental and clinical settings will be critical to optimizing outcomes.

## Declaration of interest

The authors declare that there is no conflict of interest that could be perceived as prejudicing the impartiality of the research reported.

## Funding

This work was supported by the Eunice Kennedy Shriver National Institute of Child Health and Human Developmenthttp://dx.doi.org/10.13039/100009633 of the National Institutes of Health http://dx.doi.org/10.13039/100000002 (R21HD103956); an American Society for Reproductive Medicine http://dx.doi.org/10.13039/100002603 Research Grant; and the Queenie Victorina Neri Research Scholar Award. The content is solely the responsibility of the authors and does not necessarily represent the official views of the National Institutes of Health http://dx.doi.org/10.13039/100000002 or the American Society for Reproductive Medicine http://dx.doi.org/10.13039/100002603.

## Author contribution statement

Conceptualization – L M, D J; methodology – L M, N L G, E K, L P, R B, N Z, D J; investigation – L M, N L G, D J; analysis – L M, N L G; writing – original draft preparation – L M, N L G, D J; writing – review and editing – L M, N L G, G S, Z R, D J; supervision L M, D J; project administration G S, Z R, D J; funding acquisition – Z R, D J.

## References

[bib1] Andersen CY Silber SJ Bergholdt SH Jorgensen JS Ernst E 2012 Long-term duration of function of ovarian tissue transplants: case reports. Reproductive Biomedicine Online 25 128–132. (10.1016/j.rbmo.2012.03.014) 22687323

[bib2] Anderson RA Wallace WH Baird DT 2008 Ovarian cryopreservation for fertility preservation: indications and outcomes. Reproduction 136 681–689. (10.1530/REP-08-0097) 18682546

[bib3] Aubard Y Piver P Cogni Y Fermeaux V Poulin N Driancourt MA 1999 Orthotopic and heterotopic autografts of frozen-thawed ovarian cortex in sheep. Human Reproduction 14 2149–2154. (10.1093/humrep/14.8.2149) 10438442

[bib4] Bastings L Beerendonk CC Westphal JR Massuger LF Kaal SE van Leeuwen FE Braat DD Peek R 2013 Autotransplantation of cryopreserved ovarian tissue in cancer survivors and the risk of reintroducing malignancy: a systematic review. Human Reproduction Update 19 483–506. (10.1093/humupd/dmt020) 23817363

[bib5] Broekmans FJ Soules MR Fauser BC 2009 Ovarian aging: mechanisms and clinical consequences. Endocrine Reviews 30 465–493. (10.1210/er.2009-0006) 19589949

[bib6] Dath C Van Eyck AS Dolmans MM Romeu L Delle Vigne L Donnez J Van Langendonckt A 2010 Xenotransplantation of human ovarian tissue to nude mice: comparison between four grafting sites. Human Reproduction 25 1734–1743. (10.1093/humrep/deq131) 20511300

[bib7] Demeestere I Simon P Dedeken L Moffa F Tsépélidis S Brachet C Delbaere A Devreker F Ferster A 2015 Live birth after autograft of ovarian tissue cryopreserved during childhood. Human Reproduction 30 2107–2109. (10.1093/humrep/dev128) 26062556

[bib8] Dittrich R Hackl J Lotz L Hoffmann I Beckmann MW 2015 Pregnancies and live births after 20 transplantations of cryopreserved ovarian tissue in a single center. Fertility and Sterility 103 462–468. (10.1016/j.fertnstert.2014.10.045) 25487750

[bib9] Dolmans MM Martinez-Madrid B Gadisseux E Guiot Y Yuan WY Torre A Camboni A Van Langendonckt A Donnez J 2007 Short-term transplantation of isolated human ovarian follicles and cortical tissue into nude mice. Reproduction 134 253–262. (10.1530/REP-07-0131) 17660235

[bib10] Dolmans MM von Wolff M Poirot C Diaz-Garcia C Cacciottola L Boissel N Liebenthron J Pellicer A Donnez J Andersen CY 2021 Transplantation of cryopreserved ovarian tissue in a series of 285 women: a review of five leading European centers. Fertility and Sterility 115 1102–1115. (10.1016/j.fertnstert.2021.03.008) 33933173

[bib11] Donnez J Dolmans MM 2015 Ovarian cortex transplantation: 60 reported live births brings the success and worldwide expansion of the technique towards routine clinical practice. Journal of Assisted Reproduction and Genetics 32 1167–1170. (10.1007/s10815-015-0544-9) 26210678 PMC4554373

[bib12] Donnez J Dolmans MM Demylle D Jadoul P Pirard C Squifflet J Martinez-Madrid B van Langendonckt A 2004 Livebirth after orthotopic transplantation of cryopreserved ovarian tissue. Lancet 364 1405–1410. (10.1016/S0140-6736(0417222-X) 15488215

[bib13] Donnez J Martinez-Madrid B Jadoul P Van Langendonckt A Demylle D Dolmans MM 2006 Ovarian tissue cryopreservation and transplantation: a review. Human Reproduction Update 12 519–535. (10.1093/humupd/dml032) 16849817

[bib14] Durlinger AL Visser JA Themmen AP 2002 Regulation of ovarian function: the role of anti-Mullerian hormone. Reproduction 124 601–609. (10.1530/rep.0.1240601) 12416998

[bib15] Gavish Z Peer G Roness H Cohen Y Meirow D 2015 Follicle activation and ‘burn-out’ contribute to post-transplantation follicle loss in ovarian tissue grafts: the effect of graft thickness. Human Reproduction 30 1003. (10.1093/humrep/dev020) 25712231

[bib16] Gellert SE Pors SE Kristensen SG Bay-Bjørn AM Ernst E Yding Andersen C 2018 Transplantation of frozen-thawed ovarian tissue: an update on worldwide activity published in peer-reviewed papers and on the Danish cohort. Journal of Assisted Reproduction and Genetics 35 561–570. (10.1007/s10815-018-1144-2) 29497953 PMC5949119

[bib17] Gook DA Edgar DH Borg J Archer J McBain JC 2005 Diagnostic assessment of the developmental potential of human cryopreserved ovarian tissue from multiple patients using xenografting. Human Reproduction 20 72–78. (10.1093/humrep/deh550) 15471928

[bib18] Gook D Hale L Polyakov A Manley T Rozen G Stern K 2021 Experience with transplantation of human cryopreserved ovarian tissue to a sub-peritoneal abdominal site. Human Reproduction 36 2473–2483. (10.1093/humrep/deab167) 34255039

[bib19] Gougeon A 1996 Regulation of ovarian follicular development in primates: facts and hypotheses. Endocrine Reviews 17 121–155. (10.1210/edrv-17-2-121) 8706629

[bib20] Hansen KR Knowlton NS Thyer AC Charleston JS Soules MR Klein NA 2008 A new model of reproductive aging: the decline in ovarian non-growing follicle number from birth to menopause. Human Reproduction 23 699–708. (10.1093/humrep/dem408) 18192670

[bib21] ISFP Practice Committee, Kim SS Donnez J Barri P Pellicer A Patrizio P Rosenwaks Z Nagy P Falcone T Andersen C 2012 Recommendations for fertility preservation in patients with lymphoma, leukemia, and breast cancer. Journal of Assisted Reproduction and Genetics 29 465–468. (10.1007/s10815-012-9786-y) 22648282 PMC3370045

[bib22] Jadoul P Guilmain A Squifflet J Luyckx M Votino R Wyns C Dolmans MM 2017 Efficacy of ovarian tissue cryopreservation for fertility preservation: lessons learned from 545 cases. Human Reproduction 32 1046–1054. (10.1093/humrep/dex040) 28333228

[bib23] Jensen AK Kristensen SG Macklon KT Jeppesen JV Fedder J Ernst E Andersen CY 2015 Outcomes of transplantations of cryopreserved ovarian tissue to 41 women in Denmark. Human Reproduction 30 2838–2845. (10.1093/humrep/dev230) 26443605

[bib24] Kawamura K Cheng Y Suzuki N Deguchi M Sato Y Takae S Ho CH Kawamura N Tamura M Hashimoto S 2013 Hippo signaling disruption and Akt stimulation of ovarian follicles for infertility treatment. PNAS 110 17474–17479. (10.1073/pnas.1312830110) 24082083 PMC3808580

[bib25] Kim SS 2012 Assessment of long term endocrine function after transplantation of frozen-thawed human ovarian tissue to the heterotopic site: 10 year longitudinal follow-up study. Journal of Assisted Reproduction and Genetics 29 489–493. (10.1007/s10815-012-9757-3) 22492223 PMC3370050

[bib26] Kim SS Lee WS Chung MK Lee HC Lee HH Hill D 2009 Long-term ovarian function and fertility after heterotopic autotransplantation of cryobanked human ovarian tissue: 8-year experience in cancer patients. Fertility and Sterility 91 2349–2354. (10.1016/j.fertnstert.2008.04.019) 18675964

[bib27] Krogh A 1919 The rate of diffusion of gases through animal tissues, with some remarks on the coefficient of invasion. Journal of Physiology 52 391–408. (10.1113/jphysiol.1919.sp001838) 16993404 PMC1402717

[bib28] Lass A 2004 Assessment of ovarian reserve: is there still a role for ovarian biopsy in the light of new data? Human Reproduction 19 467–469. (10.1093/humrep/deh118) 14998936

[bib29] Lotz L Dittrich R Hoffmann I Beckmann MW 2019 Ovarian tissue transplantation: experience from Germany and worldwide efficacy. Clinical Medicine Insights: Reproductive Health 13 1179558119867357. (10.1177/1179558119867357) 31431803 PMC6685107

[bib30] Man L Park L Bodine R Ginsberg M Zaninovic N Man OA Schattman G Rosenwaks Z James D 2017 Engineered endothelium provides angiogenic and paracrine stimulus to grafted human ovarian tissue. Scientific Reports 7 8203. (10.1038/s41598-017-08491-z) PMC5557862 28811567

[bib31] Man L Park L Bodine R Ginsberg M Zaninovic N Schattman G Schwartz RE Rosenwaks Z James D 2018 Co-transplantation of human ovarian tissue with engineered endothelial cells: a cell-based strategy combining accelerated perfusion with direct paracrine delivery. Journal of Visualized Experiments 135 57472. (10.3791/57472) PMC6101226 29863664

[bib32] Man L Lustgarten-Guahmich N Kallinos E Redhead-Laconte Z Liu S Schattman B Redmond D Hancock K Zaninovic N Schattman G 2020 Comparison of human antral follicles of xenograft versus ovarian origin reveals disparate molecular signatures. Cell Reports 32 108027. (10.1016/j.celrep.2020.108027) 32783948

[bib33] Man L Lustgarten Guahmich N Kallinos E Park L Caiazza B Khan M Liu ZY Patel R Torres C Lekovich J 2021 Exogenous insulin-like growth factor 1 accelerates growth and maturation of follicles in human cortical xenografts and increases ovarian output in mice. F&S Science 2 237–247. (10.1016/j.xfss.2021.07.002) 35560275 PMC9361175

[bib34] Man L Lustgarten-Guahmich N Kallinos E Caiazza B Khan M Liu ZY Patel R Torres C Pepin D Yang HS 2022 Chronic superphysiologic AMH promotes premature luteinization of antral follicles in human ovarian xenografts. Science Advances 8 eabi7315. (10.1126/sciadv.abi7315) 35263130 PMC8906729

[bib35] Matthews SJ Picton H Ernst E Andersen CY 2018 Successful pregnancy in a woman previously suffering from β-thalassemia following transplantation of ovarian tissue cryopreserved before puberty. Minerva Ginecologica 70 432–435. (10.23736/S0026-4784.18.04240-5) 29696941

[bib36] Meirow D Levron J Eldar-Geva T Hardan I Fridman E Zalel Y Schiff E Dor J 2005 Pregnancy after transplantation of cryopreserved ovarian tissue in a patient with ovarian failure after chemotherapy. New England Journal of Medicine 353 318–321. (10.1056/NEJMc055237) 15983020

[bib37] Meirow D Baum M Yaron R Levron J Hardan I Schiff E Nagler A Yehuda DB Raanani H Hourvitz A 2007 Ovarian tissue cryopreservation in hematologic malignancy: ten years’ experience. Leukemia and Lymphoma 48 1569–1576. (10.1080/10428190701471957) 17701589

[bib38] Meirow D Ra’anani H Shapira M Brenghausen M Derech Chaim S Aviel-Ronen S Amariglio N Schiff E Orvieto R Dor J 2016 Transplantations of frozen-thawed ovarian tissue demonstrate high reproductive performance and the need to revise restrictive criteria. Fertility and Sterility 106 467–474. (10.1016/j.fertnstert.2016.04.031) 27181924

[bib39] Newton H Aubard Y Rutherford A Sharma V Gosden R 1996 Low temperature storage and grafting of human ovarian tissue. Human Reproduction 11 1487–1491. (10.1093/oxfordjournals.humrep.a019423) 8671490

[bib40] Nisolle M Casanas-Roux F Qu J Motta P Donnez J 2000 Histologic and ultrastructural evaluation of fresh and frozen-thawed human ovarian xenografts in nude mice. Fertility and Sterility 74 122–129. (10.1016/s0015-0282(0000548-3) 10899508

[bib41] Oktay K 2001 Ovarian tissue cryopreservation and transplantation: preliminary findings and implications for cancer patients. Human Reproduction Update 7 526–534. (10.1093/humupd/7.6.526) 11727860

[bib42] Oktay K 2002 Evidence for limiting ovarian tissue harvesting for the purpose of transplantation to women younger than 40 years of age. Journal of Clinical Endocrinology and Metabolism 87 1907–1908. (10.1210/jcem.87.4.8367) 11932340

[bib43] Oktay K Newton H Mullan J Gosden RG 1998 Development of human primordial follicles to antral stages in SCID/hpg mice stimulated with follicle stimulating hormone. Human Reproduction 13 1133–1138. (10.1093/humrep/13.5.1133) 9647533

[bib44] Oktay K Economos K Kan M Rucinski J Veeck L Rosenwaks Z 2001 Endocrine function and oocyte retrieval after autologous transplantation of ovarian cortical strips to the forearm. JAMA 286 1490–1493. (10.1001/jama.286.12.1490) 11572742

[bib45] Oktay K Buyuk E Veeck L Zaninovic N Xu K Takeuchi T Opsahl M Rosenwaks Z 2004 Embryo development after heterotopic transplantation of cryopreserved ovarian tissue. Lancet 363 837–840. (10.1016/S0140-6736(0415728-0) 15031026

[bib46] Poirot C Vacher-Lavenu MC Helardot P Guibert J Brugières L Jouannet P 2002 Human ovarian tissue cryopreservation: indications and feasibility. Human Reproduction 17 1447–1452. (10.1093/humrep/17.6.1447) 12042259

[bib47] Poirot C Brugieres L Yakouben K Prades-Borio M Marzouk F de Lambert G Pacquement H Bernaudin F Neven B Paye-Jaouen A 2019 Ovarian tissue cryopreservation for fertility preservation in 418 girls and adolescents up to 15 years of age facing highly gonadotoxic treatment. Twenty years of experience at a single center. Acta Obstetricia et Gynecologica Scandinavica 98 630–637. (10.1111/aogs.13616) 30919447

[bib48] Practice Committee of the American Society for Reproductive Medicine. Electronic address: asrm@asrm.org 2019 Fertility preservation in patients undergoing gonadotoxic therapy or gonadectomy: a committee opinion. Fertility and Sterility 112 1022–1033. (10.1016/j.fertnstert.2019.09.013) 31843073

[bib49] Qu J Godin PA Nisolle M Donnez J 2000 Distribution and epidermal growth factor receptor expression of primordial follicles in human ovarian tissue before and after cryopreservation. Human Reproduction 15 302–310. (10.1093/humrep/15.2.302) 10655299

[bib50] Roness H Meirow D 2019 FERTILITY PRESERVATION: Follicle reserve loss in ovarian tissue transplantation. Reproduction 158 F35–F44. (10.1530/REP-19-0097) 31394506

[bib51] Ruan X Cui Y Du J Jin J Gu M Chen S Mueck AO 2019 Randomized study to prove the quality of human ovarian tissue cryopreservation by xenotransplantation into mice. Journal of Ovarian Research 12 46. (10.1186/s13048-019-0521-5) PMC6530171 31113493

[bib52] Schmidt KL Byskov AG Nyboe Andersen A Muller J Yding Andersen C 2003 Density and distribution of primordial follicles in single pieces of cortex from 21 patients and in individual pieces of cortex from three entire human ovaries. Human Reproduction 18 1158–1164. (10.1093/humrep/deg246) 12773440

[bib53] Seandel M Butler JM Kobayashi H Hooper AT White IA Zhang F Vertes EL Kobayashi M Zhang Y Shmelkov SV 2008 Generation of a functional and durable vascular niche by the adenoviral E4ORF1 gene. PNAS 105 19288–19293. (10.1073/pnas.0805980105) 19036927 PMC2588414

[bib54] Shapira M Dolmans MM Silber S Meirow D 2020 Evaluation of ovarian tissue transplantation: results from three clinical centers. Fertility and Sterility 114 388–397. (10.1016/j.fertnstert.2020.03.037) 32605799

[bib55] Shultz LD Lyons BL Burzenski LM Gott B Chen X Chaleff S Kotb M Gillies SD King M Mangada J 2005 Human lymphoid and myeloid cell development in NOD/LtSz-scid IL2R gamma null mice engrafted with mobilized human hemopoietic stem cells. Journal of Immunology 174 6477–6489. (10.4049/jimmunol.174.10.6477) 15879151

[bib56] Soleimani R De Vos W Van Oostveldt P Lierman S Van den Broecke R De Sutter P Dhont M Van der Elst J 2006 Two novel techniques to detect follicles in human ovarian cortical tissue. Human Reproduction 21 1720–1724. (10.1093/humrep/del057) 16517556

[bib57] Soleimani R Heytens E Van den Broecke R Rottiers I Dhont M Cuvelier CA De Sutter P 2010 Xenotransplantation of cryopreserved human ovarian tissue into murine back muscle. Human Reproduction 25 1458–1470. (10.1093/humrep/deq055) 20299384

[bib58] Sonmezer M Oktay K 2010 Orthotopic and heterotopic ovarian tissue transplantation. Best Practice and Research: Clinical Obstetrics and Gynaecology 24 113–126. (10.1016/j.bpobgyn.2009.09.002) 19853515

[bib59] Stern CJ Gook D Hale LG Agresta F Oldham J Rozen G Jobling T 2014 Delivery of twins following heterotopic grafting of frozen-thawed ovarian tissue. Human Reproduction 29 1828. (10.1093/humrep/deu119) 24871346

[bib60] Stoop D Cobo A Silber S 2014 Fertility preservation for age-related fertility decline. Lancet 384 1311–1319. (10.1016/S0140-6736(1461261-7) 25283572

[bib61] Van der Ven H Liebenthron J Beckmann M Toth B Korell M Krüssel J Frambach T Kupka M Hohl MK Winkler-Crepaz K 2016 Ninety-five orthotopic transplantations in 74 women of ovarian tissue after cytotoxic treatment in a fertility preservation network: tissue activity, pregnancy and delivery rates. Human Reproduction 31 2031–2041. (10.1093/humrep/dew165) 27378768

[bib62] Van Eyck AS Jordan BF Gallez B Heilier JF Van Langendonckt A Donnez J 2009 Electron paramagnetic resonance as a tool to evaluate human ovarian tissue reoxygenation after xenografting. Fertility and Sterility 92 374–381. (10.1016/j.fertnstert.2008.05.012) 18692811

[bib63] Wallace WH Smith AG Kelsey TW Edgar AE Anderson RA 2014 Fertility preservation for girls and young women with cancer: population-based validation of criteria for ovarian tissue cryopreservation. Lancet: Oncology 15 1129–1136. (10.1016/S1470-2045(1470334-1) 25130994 PMC4153375

[bib64] Weenen C Laven JS Von Bergh AR Cranfield M Groome NP Visser JA Kramer P Fauser BC Themmen AP 2004 Anti-Mullerian hormone expression pattern in the human ovary: potential implications for initial and cyclic follicle recruitment. Molecular Human Reproduction 10 77–83. (10.1093/molehr/gah015) 14742691

[bib65] Weissman A Gotlieb L Colgan T Jurisicova A Greenblatt EM Casper RF 1999 Preliminary experience with subcutaneous human ovarian cortex transplantation in the NOD-SCID mouse. Biology of Reproduction 60 1462–1467. (10.1095/biolreprod60.6.1462) 10330106

[bib66] Woodruff TK Shea LD 2011 A new hypothesis regarding ovarian follicle development: ovarian rigidity as a regulator of selection and health. Journal of Assisted Reproduction and Genetics 28 3–6. (10.1007/s10815-010-9478-4) 20872066 PMC3045494

